# *N*-[(*E*,*Z*)-1,3-Diphenyl­prop-2-enyl­idene]-*N*′-(1,3-dithio­lan-2-yl­idene)hydrazine

**DOI:** 10.1107/S1600536808018606

**Published:** 2008-06-25

**Authors:** Jian-Feng Liu, Xiao-Lan Liu, Yong-Hong Liu

**Affiliations:** a Technology Center, Jiuquan Iron and Steel (Group) Co. Ltd, Jiayuguan 735100, People’s Republic of China; b College of Chemistry and Chemical Engineering, Yangzhou University, Yangzhou 225002, People’s Republic of China

## Abstract

Mol­ecules of the title compound, C_18_H_16_N_2_S_2_, exist as the (2*E*, 1′*Z*)-isomer. The 1,3-dithiol­ane ring has an envelope conformation; the atoms of the C—C bond are disordered over two positions with occupancies of 0.47 (7) and 0.53 (7). The structure exhibits inter­molecular C—H⋯S and C—H⋯π(arene) hydrogen bonds.

## Related literature

For related literature, see: Beghidja *et al.* (2006 ▶); Cremer & Pople (1975 ▶); Gou *et al.* (2004 ▶); Liu *et al.* (2001 ▶, 2003 ▶, 2007 ▶, 2008 ▶); Wang *et al.* (1994 ▶); Xu *et al.* (2005 ▶); Yang *et al.* (2007 ▶); Yarishkin *et al.* (2008 ▶); Zhai *et al.* (1999 ▶).
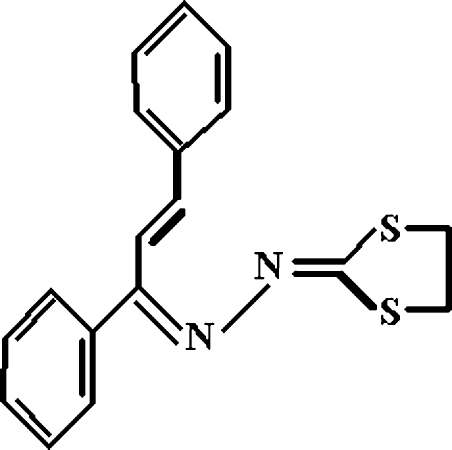


## Experimental

### 

#### Crystal data

C_18_H_16_N_2_S_2_
*M* *_r_* = 324.47
Orthorhombic, 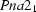

*a* = 30.9008 (9) Å
*b* = 5.7352 (2) Å
*c* = 9.1499 (3) Å
*V* = 1621.57 (9) Å^3^
*Z* = 4
Mo *K*α radiation
μ = 0.33 mm^−1^
*T* = 296 (2) K
0.30 × 0.30 × 0.20 mm

#### Data collection

Bruker SMART 1000 CCD diffractometer
Absorption correction: multi-scan (*SADABS*; Bruker, 2007 ▶) *T*_min_ = 0.909, *T*_max_ = 0.938
7320 measured reflections
3339 independent reflections
2833 reflections with *I* > 2σ(*I*)
*R*_int_ = 0.055

#### Refinement

*R*[*F* ^2^ > 2σ(*F* ^2^)] = 0.046
*wR*(*F* ^2^) = 0.117
*S* = 1.00
3339 reflections
206 parameters
7 restraints
H-atom parameters constrained
Δρ_max_ = 0.47 e Å^−3^
Δρ_min_ = −0.34 e Å^−3^
Absolute structure: Flack (1983 ▶), 1186 Friedel pairs
Flack parameter: −0.03 (9)

### 

Data collection: *SMART* (Bruker, 2007 ▶); cell refinement: *SAINT* (Bruker, 2007 ▶); data reduction: *SAINT*; program(s) used to solve structure: *SHELXS97* (Sheldrick, 2008 ▶); program(s) used to refine structure: *SHELXL97* (Sheldrick, 2008 ▶); molecular graphics: *PLATON* (Spek, 2003 ▶); software used to prepare material for publication: *SHELXTL* (Sheldrick, 2008 ▶).

## Supplementary Material

Crystal structure: contains datablocks I, global. DOI: 10.1107/S1600536808018606/om2242sup1.cif

Structure factors: contains datablocks I. DOI: 10.1107/S1600536808018606/om2242Isup2.hkl

Additional supplementary materials:  crystallographic information; 3D view; checkCIF report

## Figures and Tables

**Table 1:** Hydrogen-bond geometry (Å, °)

*D*—H⋯*A*	*D*—H	H⋯*A*	*D*⋯*A*	*D*—H⋯*A*
C17′—H17*B*⋯*Cg*^i^	0.97	2.94	3.842 (9)	156
C18′—H18*A*⋯S1^ii^	0.97	2.69	3.439 (6)	135
C18′—H18*B*⋯S1^i^	0.97	2.86	3.628 (7)	137

Symmetry codes: (i) 
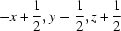
; (ii) 
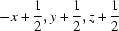
. *Cg* is the centroid of atoms C10–C15.

**Fig. 1.**
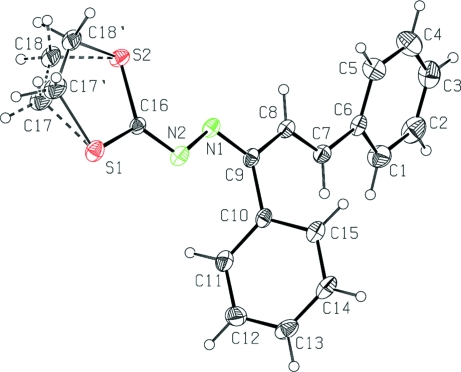
The molecular structure of the title compound, showing 30% probability ellipsoids. Open bonds show the minor disorder component.

**Fig. 2.**
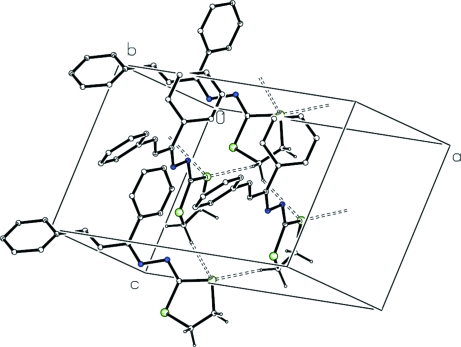
Part of the crystal structure, showing C–H···S inter-molecular hydrogen bonds as dashed lines. For the sake of clarity, H atoms not involved in hydrogen bonding have been omitted.

**Fig. 3.**
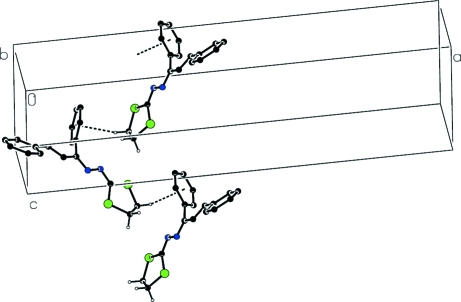
Part of the crystal structure, showing C–H···π as dashed lines. For the sake of clarity, H atoms not involved in hydrogen bonding have been omitted.

## supplementary crystallographic information

### Comment

The Schiff bases and carbonyl derivatives of 2-hydrazono-1, 3-dithiolane have been abstracted for their coordination chemistry and biological activity (Beghidja *et al.*, 2006; Wang *et al.*, 1994, Gou *et al.*, 2004; Xu *et al.*, 2005). Chalcone and its derivatives, as a natural products, have shown strong antibacterial, antifungal, antitumor and anti-inflammatory properties, especially antileishmanial, antimalarial and antimalarial (Zhai *et al.*, 1999; Liu *et al.*, 2001, 2003). Some chalcones demonstrated the ability to block voltage-dependent potassium channels (Yarishkin *et al.*, 2008). As ongoing research (Liu *et al.*, 2008, Yang *et al.*, 2007), we report herein the synthesis and structure ofthe title compound *via *the condensation reaction of the simple chalcone, (*E*)-1,3-diphenyl-propenone, and 2-hydrazono-1, 3-dithiolane.

The molecule of the title compound exists as the most stable configuration of (2*E, 1'Z)*-isomer (Fig.1). In the molecule the atoms of C16, N2, N1, C9, C8, C7 and two phenyl rings form a large conjugated system, but only the atoms of S1, S2, C16, N2, N1, C9 and C8 are coplanar, and C7, C6 and C10 are deviate by 0.22, 0.24, 0.11 (2) Å from the plane, respectively. The dihedral angles between the plane to phenyl ring (C10 ~ C15) plane and (C1~C6) plane are 63.45 (2)° and 43.30 (18)°, respectively. The two phenyl ring planes are almost vertical (the dihedral angle between them is 84.16°) (Fig. 1, Table 2).

In the ring of 1, 3-dithiolane of the molecule, the atoms of C17 and C18 are disordered over two positions with relative occupancies of 0.43 (7):0.57 (7) for the minor and major components. The rings at the two positions are also in the envelope (Cremer *et al.*, 1975) form, and atoms C18 and C18' respectively deviate by - 0.367 (3) Å and 0.402 (2) Å from the plane defined by S1, C16 and S2, just like that in (3*E*)-3-(1, 3-dithiolan-2-ylidenehydrazono)buton (Liu *et al.*, 2007).

In its packing structure, the molecules are linked into a three-dimensional framework by C–H···S and C–H···π(arene) intermolecular hydrogen bonds in the major components (Fig. 2 and 3, Table 2).

### Experimental

The title compound was prepared from (E)-1, 3-diphenyl-propenone and 2-hydrazono-1, 3-dithiolane in the equimolar ratio in 95% EtOH. The resulting yellow solid was recrystallized from CH_2_Cl_2_–EtOH to give crystals of suitable for single-crystal X-ray diffraction (yield 88%, m.p. 440–442 K). Analysis calculated for C_18_H_16_N_2_S_2_: C, 66.63; H, 4.97; N, 8.63*%*; found: C, 66.36; H, 4.81; N, 8.28.^1^H NMR (600 MHz, CDCl_3_, δ, p.p.m.): 7.879(d, J=16.47 Hz, 1H, CH=C), 6.891(d, J=16.54 Hz, 1H, CH=C), 7.324~7.677(m, 10H, 2C_6_H_5_), 3.521(m, 2H, CH_2_), 3.460(m, 2H, CH_2_).

### Refinement

The C17 and C18 atoms were refined as disordered with refined occupancy of 57.3 (7) % for the major component. The anisotropic displacement parameters of C17, C17', C18 and C18' were constrained to be equal. After their location in a difference map, all H atoms were fixed geometrically at ideal positions and allowed to ride on the parent C atoms, with C — H distances of 0.93 (aromatic and ethylenic CH) or 0.97 Å (methene), and with *U_iso_*(H) values of *1.2U_eq_* (C).

### Crystal data

**Table:**

C_18_H_16_N_2_S_2_	*D*_x_ = 1.329 Mg m^−^^3^
*M**_r_* = 324.47	Melting point: 442 K
Orthorhombic, *P**n**a*2_1_	Mo *K*α radiation, λ = 0.71073 Å
Hall symbol: P 2c -2n	Cell parameters from 2883 reflections
*a* = 30.9008 (9) Å	θ = 2.6–28.1°
*b* = 5.7352 (2) Å	µ = 0.33 mm^−^^1^
*c* = 9.1499 (3) Å	*T* = 296 K
*V* = 1621.57 (9) Å^3^	Block, yellow
*Z* = 4	0.30 × 0.30 × 0.20 mm
*F*(000) = 680	

### Data collection

**Table:**

Bruker SMART 1000 CCD diffractometer	3339 independent reflections
Radiation source: fine-focus sealed tube	2833 reflections with *I* > 2σ(*I*)
graphite	*R*_int_ = 0.055
ω scans	θ_max_ = 28.4°, θ_min_ = 2.6°
Absorption correction: multi-scan (*SADABS*; Bruker, 2007)	*h* = −41→39
*T*_min_ = 0.909, *T*_max_ = 0.938	*k* = −4→7
7320 measured reflections	*l* = −12→11

### Refinement

**Table:**

Refinement on *F*^2^	Secondary atom site location: difference Fourier map
Least-squares matrix: full	Hydrogen site location: inferred from neighbouring sites
*R*[*F*^2^ > 2σ(*F*^2^)] = 0.046	H-atom parameters constrained
*wR*(*F*^2^) = 0.117	*w* = 1/[σ^2^(*F*_o_^2^) + (0.0727*P*)^2^] where *P* = (*F*_o_^2^ + 2*F*_c_^2^)/3
*S* = 1.00	(Δ/σ)_max_ = 0.003
3339 reflections	Δρ_max_ = 0.47 e Å^−^^3^
206 parameters	Δρ_min_ = −0.34 e Å^−^^3^
7 restraints	Absolute structure: Flack (1983), 1186 Friedel pairs
Primary atom site location: structure-invariant direct methods	Flack parameter: −0.03 (9)

### Special details

**Table:**

Geometry. All e.s.d.'s (except the e.s.d. in the dihedral angle between two l.s. planes) are estimated using the full covariance matrix. The cell e.s.d.'s are taken into account individually in the estimation of e.s.d.'s in distances, angles and torsion angles; correlations between e.s.d.'s in cell parameters are only used when they are defined by crystal symmetry. An approximate (isotropic) treatment of cell e.s.d.'s is used for estimating e.s.d.'s involving l.s. planes.
Refinement. Refinement of *F*^2^ against ALL reflections. The weighted *R*-factor *wR* and goodness of fit *S* are based on *F*^2^, conventional *R*-factors *R* are based on *F*, with *F* set to zero for negative *F*^2^. The threshold expression of *F*^2^ > σ(*F*^2^) is used only for calculating *R*-factors(gt) *etc*. and is not relevant to the choice of reflections for refinement. *R*-factors based on *F*^2^ are statistically about twice as large as those based on *F*, and *R*- factors based on ALL data will be even larger.

### Fractional atomic coordinates and isotropic or equivalent isotropic displacement parameters (Å^2^)

**Table:**

	*x*	*y*	*z*	*U*_iso_*/*U*_eq_	Occ. (<1)
C1	0.00882 (11)	1.1023 (6)	0.9703 (5)	0.0507 (8)	
H1	−0.0016	1.0304	0.8863	0.061*	
C2	−0.01559 (12)	1.2737 (7)	1.0391 (5)	0.0569 (10)	
H2	−0.0428	1.3112	1.0024	0.068*	
C3	−0.00045 (12)	1.3879 (6)	1.1592 (4)	0.0526 (9)	
H3	−0.0168	1.5053	1.2025	0.063*	
C4	0.03941 (11)	1.3275 (7)	1.2162 (5)	0.0547 (9)	
H4	0.0500	1.4039	1.2985	0.066*	
C5	0.06363 (10)	1.1535 (6)	1.1509 (4)	0.0423 (7)	
H5	0.0903	1.1133	1.1908	0.051*	
C6	0.04918 (9)	1.0381 (5)	1.0281 (3)	0.0321 (6)	
C7	0.07453 (9)	0.8558 (5)	0.9542 (3)	0.0318 (6)	
H7	0.0679	0.8263	0.8568	0.038*	
C8	0.10609 (9)	0.7288 (5)	1.0139 (3)	0.0302 (6)	
H8	0.1126	0.7561	1.1117	0.036*	
C9	0.13112 (8)	0.5516 (5)	0.9387 (3)	0.0288 (6)	
C10	0.13030 (9)	0.5395 (5)	0.7762 (3)	0.0287 (6)	
C11	0.11505 (11)	0.3437 (6)	0.7030 (4)	0.0400 (7)	
H11	0.1039	0.2189	0.7559	0.048*	
C12	0.11627 (13)	0.3328 (7)	0.5522 (4)	0.0485 (9)	
H12	0.1059	0.2011	0.5044	0.058*	
C13	0.13278 (10)	0.5157 (6)	0.4720 (4)	0.0457 (8)	
H13	0.1341	0.5062	0.3706	0.055*	
C14	0.14728 (10)	0.7122 (6)	0.5424 (4)	0.0424 (8)	
H14	0.1581	0.8365	0.4883	0.051*	
C15	0.14592 (9)	0.7269 (6)	0.6941 (3)	0.0352 (7)	
H15	0.1554	0.8615	0.7408	0.042*	
C16	0.19817 (8)	0.1107 (5)	1.0334 (3)	0.0267 (6)	
C17	0.2416 (6)	−0.261 (2)	1.1233 (8)	0.061 (3)	0.427 (7)
H17C	0.2319	−0.4193	1.1064	0.073*	0.427 (7)
H17D	0.2724	−0.2661	1.1438	0.073*	0.427 (7)
C18	0.2200 (3)	−0.1717 (16)	1.2466 (9)	0.0409 (13)	0.427 (7)
H18C	0.2410	−0.1558	1.3248	0.049*	0.427 (7)
H18D	0.1989	−0.2864	1.2778	0.049*	0.427 (7)
C17'	0.2517 (3)	−0.1970 (15)	1.1350 (5)	0.061 (3)	0.573 (7)
H17A	0.2432	−0.3591	1.1449	0.073*	0.573 (7)
H17B	0.2830	−0.1927	1.1340	0.073*	0.573 (7)
C18'	0.2375 (2)	−0.0777 (12)	1.2611 (6)	0.0409 (13)	0.573 (7)
H18A	0.2612	0.0151	1.2993	0.049*	0.573 (7)
H18B	0.2295	−0.1906	1.3353	0.049*	0.573 (7)
N1	0.15384 (7)	0.4150 (4)	1.0215 (3)	0.0303 (5)	
N2	0.17893 (8)	0.2525 (4)	0.9464 (3)	0.0326 (5)	
S1	0.23331 (2)	−0.09801 (13)	0.96398 (8)	0.0371 (2)	
S2	0.19208 (2)	0.10941 (13)	1.22517 (8)	0.03412 (18)	

### Atomic displacement parameters (Å^2^)

**Table:**

	*U*^11^	*U*^22^	*U*^33^	*U*^12^	*U*^13^	*U*^23^
C1	0.0449 (17)	0.054 (2)	0.0529 (19)	0.0162 (16)	−0.0127 (17)	−0.0014 (18)
C2	0.0450 (18)	0.064 (3)	0.062 (2)	0.025 (2)	−0.0003 (18)	0.011 (2)
C3	0.0468 (19)	0.048 (2)	0.063 (2)	0.0138 (16)	0.0177 (17)	0.0037 (17)
C4	0.0448 (17)	0.057 (2)	0.062 (2)	0.0032 (17)	0.0105 (19)	−0.014 (2)
C5	0.0282 (14)	0.0498 (19)	0.0489 (19)	0.0002 (14)	0.0035 (13)	−0.0052 (15)
C6	0.0304 (13)	0.0307 (15)	0.0353 (14)	0.0037 (12)	0.0044 (11)	0.0105 (12)
C7	0.0307 (13)	0.0326 (14)	0.0321 (13)	−0.0017 (12)	−0.0014 (12)	0.0042 (13)
C8	0.0300 (12)	0.0319 (15)	0.0287 (13)	0.0000 (12)	0.0000 (10)	0.0062 (11)
C9	0.0245 (12)	0.0292 (14)	0.0326 (14)	−0.0031 (11)	0.0000 (10)	0.0083 (11)
C10	0.0256 (12)	0.0277 (14)	0.0328 (13)	0.0085 (11)	0.0001 (11)	0.0060 (11)
C11	0.0465 (16)	0.0330 (16)	0.0405 (18)	0.0057 (14)	−0.0028 (14)	0.0068 (14)
C12	0.061 (2)	0.0419 (19)	0.0426 (18)	0.0144 (18)	−0.0111 (16)	−0.0071 (15)
C13	0.0507 (18)	0.056 (2)	0.0300 (14)	0.0256 (17)	0.0009 (16)	0.0032 (16)
C14	0.0417 (16)	0.050 (2)	0.0358 (16)	0.0093 (16)	0.0069 (14)	0.0178 (15)
C15	0.0303 (13)	0.0381 (17)	0.0373 (16)	0.0033 (13)	0.0026 (11)	0.0097 (13)
C16	0.0260 (12)	0.0276 (15)	0.0266 (12)	−0.0049 (11)	−0.0006 (10)	0.0055 (11)
C17	0.087 (6)	0.050 (6)	0.046 (2)	0.039 (5)	−0.018 (3)	−0.001 (3)
C18	0.038 (3)	0.039 (4)	0.046 (3)	0.009 (2)	−0.016 (2)	0.008 (3)
C17'	0.087 (6)	0.050 (6)	0.046 (2)	0.039 (5)	−0.018 (3)	−0.001 (3)
C18'	0.038 (3)	0.039 (4)	0.046 (3)	0.009 (2)	−0.016 (2)	0.008 (3)
N1	0.0260 (11)	0.0347 (13)	0.0302 (11)	0.0046 (10)	0.0023 (9)	0.0081 (10)
N2	0.0315 (11)	0.0357 (13)	0.0306 (12)	0.0058 (10)	0.0038 (9)	0.0078 (10)
S1	0.0372 (4)	0.0369 (4)	0.0373 (4)	0.0088 (3)	0.0046 (3)	0.0062 (4)
S2	0.0336 (3)	0.0405 (4)	0.0283 (3)	0.0076 (3)	−0.0037 (3)	0.0034 (3)

### Geometric parameters (Å, °)

**Table:**

C1—C2	1.389 (5)	C13—C14	1.373 (5)
C1—C6	1.404 (4)	C13—H13	0.9300
C1—H1	0.9300	C14—C15	1.391 (4)
C2—C3	1.362 (6)	C14—H14	0.9300
C2—H2	0.9300	C15—H15	0.9300
C3—C4	1.381 (5)	C16—N2	1.284 (3)
C3—H3	0.9300	C16—S1	1.736 (3)
C4—C5	1.383 (5)	C16—S2	1.765 (3)
C4—H4	0.9300	C17—C18	1.406 (5)
C5—C6	1.378 (4)	C17—S1	1.750 (5)
C5—H5	0.9300	C17—H17C	0.9700
C6—C7	1.471 (4)	C17—H17D	0.9700
C7—C8	1.334 (4)	C18—S2	1.839 (8)
C7—H7	0.9300	C18—H18C	0.9700
C8—C9	1.451 (4)	C18—H18D	0.9700
C8—H8	0.9300	C17'—C18'	1.410 (5)
C9—N1	1.296 (3)	C17'—S1	1.759 (4)
C9—C10	1.489 (4)	C17'—H17A	0.9700
C10—C11	1.390 (5)	C17'—H17B	0.9700
C10—C15	1.397 (4)	C18'—S2	1.798 (5)
C11—C12	1.382 (5)	C18'—H18A	0.9700
C11—H11	0.9300	C18'—H18B	0.9700
C12—C13	1.378 (5)	N1—N2	1.394 (3)
C12—H12	0.9300		
			
C2—C1—C6	119.8 (4)	C13—C14—C15	120.5 (3)
C2—C1—H1	120.1	C13—C14—H14	119.7
C6—C1—H1	120.1	C15—C14—H14	119.7
C3—C2—C1	121.3 (3)	C14—C15—C10	120.0 (3)
C3—C2—H2	119.4	C14—C15—H15	120.0
C1—C2—H2	119.4	C10—C15—H15	120.0
C2—C3—C4	119.3 (3)	N2—C16—S1	120.0 (2)
C2—C3—H3	120.3	N2—C16—S2	124.7 (2)
C4—C3—H3	120.3	S1—C16—S2	115.30 (15)
C3—C4—C5	120.0 (4)	C18—C17—S1	113.9 (6)
C3—C4—H4	120.0	C18—C17—H17C	108.8
C5—C4—H4	120.0	S1—C17—H17C	108.8
C6—C5—C4	121.5 (3)	C18—C17—H17D	108.8
C6—C5—H5	119.2	S1—C17—H17D	108.8
C4—C5—H5	119.2	H17C—C17—H17D	107.7
C5—C6—C1	118.0 (3)	C17—C18—S2	117.1 (6)
C5—C6—C7	122.9 (3)	C17—C18—H18C	108.0
C1—C6—C7	119.1 (3)	S2—C18—H18C	108.0
C8—C7—C6	126.1 (3)	C17—C18—H18D	108.0
C8—C7—H7	117.0	S2—C18—H18D	108.0
C6—C7—H7	117.0	H18C—C18—H18D	107.3
C7—C8—C9	125.3 (3)	C18'—C17'—S1	118.1 (5)
C7—C8—H8	117.3	C18'—C17'—H17A	107.8
C9—C8—H8	117.3	S1—C17'—H17A	107.8
N1—C9—C8	115.8 (2)	C18'—C17'—H17B	107.8
N1—C9—C10	124.4 (3)	S1—C17'—H17B	107.8
C8—C9—C10	119.8 (2)	H17A—C17'—H17B	107.1
C11—C10—C15	118.6 (3)	C17'—C18'—S2	112.4 (5)
C11—C10—C9	121.7 (3)	C17'—C18'—H18A	109.1
C15—C10—C9	119.7 (3)	S2—C18'—H18A	109.1
C12—C11—C10	120.6 (4)	C17'—C18'—H18B	109.1
C12—C11—H11	119.7	S2—C18'—H18B	109.1
C10—C11—H11	119.7	H18A—C18'—H18B	107.8
C13—C12—C11	120.5 (4)	C9—N1—N2	114.7 (2)
C13—C12—H12	119.8	C16—N2—N1	112.1 (2)
C11—C12—H12	119.8	C16—S1—C17	98.9 (3)
C14—C13—C12	119.7 (3)	C16—S1—C17'	95.7 (3)
C14—C13—H13	120.1	C16—S2—C18'	95.8 (2)
C12—C13—H13	120.1	C16—S2—C18	93.4 (3)

### Hydrogen-bond geometry (Å, °)

**Table:**

*D*—H···*A*	*D*—H	H···*A*	*D*···*A*	*D*—H···*A*
C17'—H17B···Cg^i^	0.97	2.94	3.842 (9)	156
C18'—H18A···S1^ii^	0.97	2.69	3.439 (6)	135
C18'—H18B···S1^i^	0.97	2.86	3.628 (7)	137

Symmetry codes: (i) −*x*+1/2, *y*−1/2, *z*+1/2; (ii) −*x*+1/2, *y*+1/2, *z*+1/2.
